# Supplementary material for: Modeling spatial, developmental, physiological, and topological constraints on human brain connectivity
Source: Sci Adv. 2022 Jun 3;8(22):eabm6127. doi: 10.1126/sciadv.abm6127 (PMC9166341; doi:10.1126/sciadv.abm6127)
Supplement: Supplementary file 1 — Supplementary Text Figs. S1 to S9 [file sciadv.abm6127_sm.pdf]

Science Advances
AAAS

Supplementary Materials for

Modeling spatial, developmental, physiological, and topological constraints on human brain connectivity

Stuart Oldham *et al.*

Corresponding author: Stuart Oldham, stuart.oldham@monash.edu

Sci. Adv. **8**, eabm6127 (2022)
DOI: 10.1126/sciadv.abm6127

This PDF file includes:

Supplementary Text
Figs. S1 to S9

Supplementary Text

To validate our new additive formulation of the cost-topology trade-off (Eq. 2) model, we compared its performance to the classical multiplicative form (Eq. 1). We first considered the additive formulation without the γ parameter (i.e., without non-linear scaling of the topology term). Under this additive formulation, 10 of the 12 trade-off models, all of which are based on degree and clustering, show comparable performance to the spatial model. Nearly all of these 10 models had $\alpha \approx 0$ (except for the clu-min, clu-prod, and deg-min models, where $\alpha < 1$), indicating that variations in topology have minimal influence on model performance and that wiring is largely determined by spatial constraints. The two additive trade-off models that perform better than the spatial model correspond to the homophilic matching and neighbor models. Critically, the mean F_{CV} of the additive matching index models ($F_{CV} = 0.23 \pm 0.01$) is significantly lower than the multiplicative matching index model ($F_{CV} = 0.27 \pm 0.01$, $p_{FWER}(325) < .05$); it is also significantly lower than the multiplicative clu-avg model which was the best-fitting multiplicative model on average ($F_{CV} = 0.26 \pm 0.02$, $p_{FWER}(325) < .05$; fig. S1A). Thus, when considering the best-fitting models, the additive formulation offers a more accurate representation of the data than the multiplicative formulation.

Examining the types of edges formed by the empirical and best fitting networks, the additive matching model indicates that the most notable improvement is in capturing mid- to long-range connections when compared to its multiplicative counterpart (fig. S2). This result confirms our intuition that the additive formulation is more effective in trading-off topological value with distance in determining connection probabilities.

We next evaluated whether an additional non-linear scaling of the topology term T_{ij} improves model performance. To this end, we compared the performance of additive models with and without the scaling exponent γ for static models. We found that the non-linear scaling improves the fit of nearly all degree and clustering based trade-off models relative to strictly linear variants of the additive models, however homophily models saw no clear benefit (fig. S1B). The best additive models also showed clear improvement over the multiplicative ones (fig. S1C). In the growth form, homophily models showed a clear improvement when the additive model included a γ scaling parameter as compared to when γ was not included (fig. S3). The superior performance of the additive model with the γ parameter suggests that the influence of topology on connection probabilities requires some non-linear scaling, especially when geometric growth is

being incorporated into the model, such that regions with a high topology score (or low if γ is negative) are disproportionately more likely to form a connection.

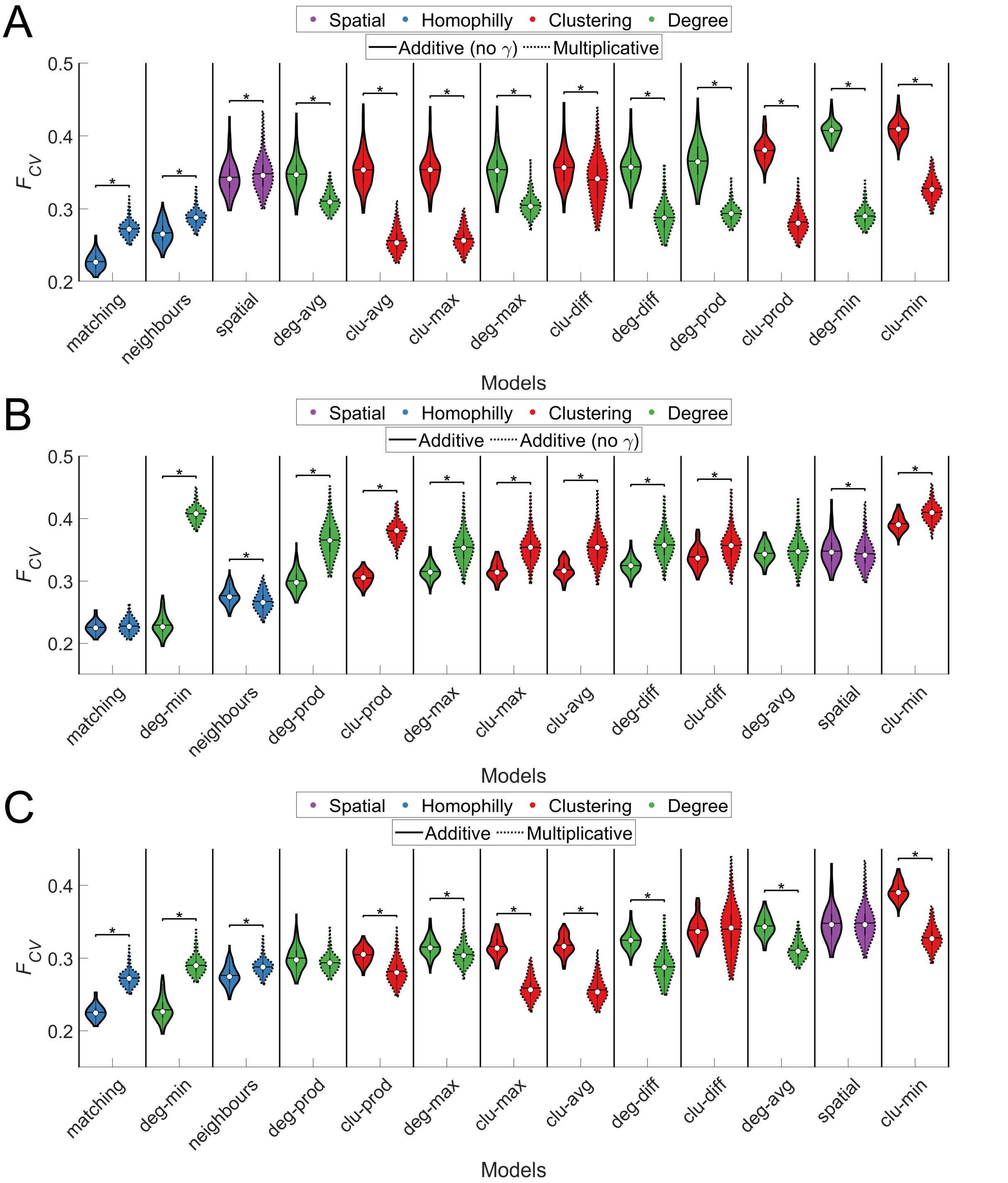

Fig. S1. **Cross-validated F_{CV} values for static generative models.** The color of each violin plot indicates the topology metric used in the model: homophily is shown in blue, clustering in red, degree in green, communicability in orange, and geometric in purple. The white circle indicates the median of each distribution, while the horizontal black line indicates the mean. **(A)** F_{CV} values for the additive (with no γ parameter) and multiplicative model formulations. **(B)** Comparison of F_{CV} for the additive and additive (with no γ parameter) formulations. **(C)** F_{CV} values for the additive and multiplicative model formulations. * $p_{FWER}(325) < .05$ (Wilcoxon signed-rank test, Bonferroni corrected for all 325 tests between all 26 models). The additive matching model achieves the best performance.

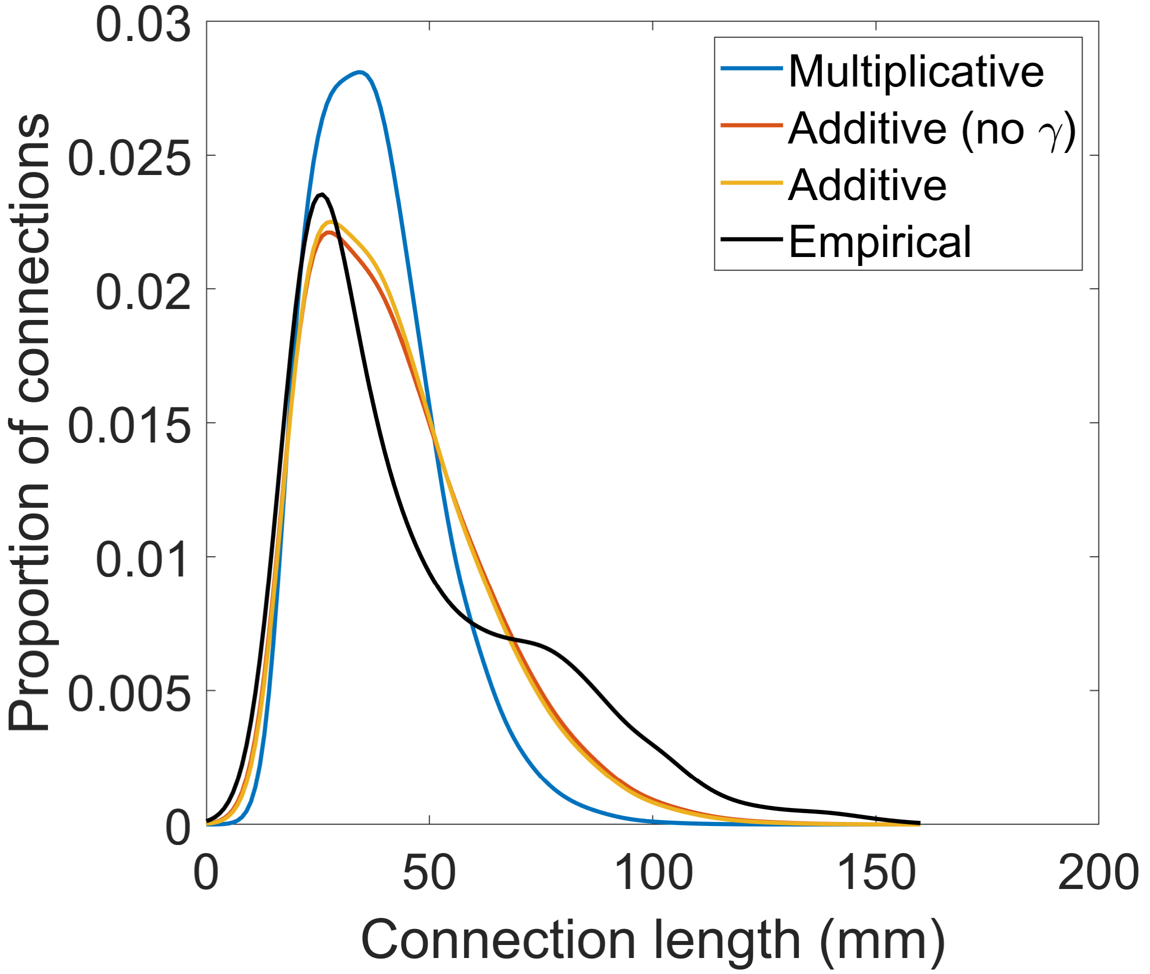

Fig. S2. **Kernel density plot of connection lengths for matching index models.** For each participant, we generated 100 networks using their optimal parameters for the matching model. For each of these networks, the kernel density of its edge lengths was calculated and then averaged. This was done for each model formulation. Additive formulations more closely match the empirical data, and are better able to reproduce long-range connections.

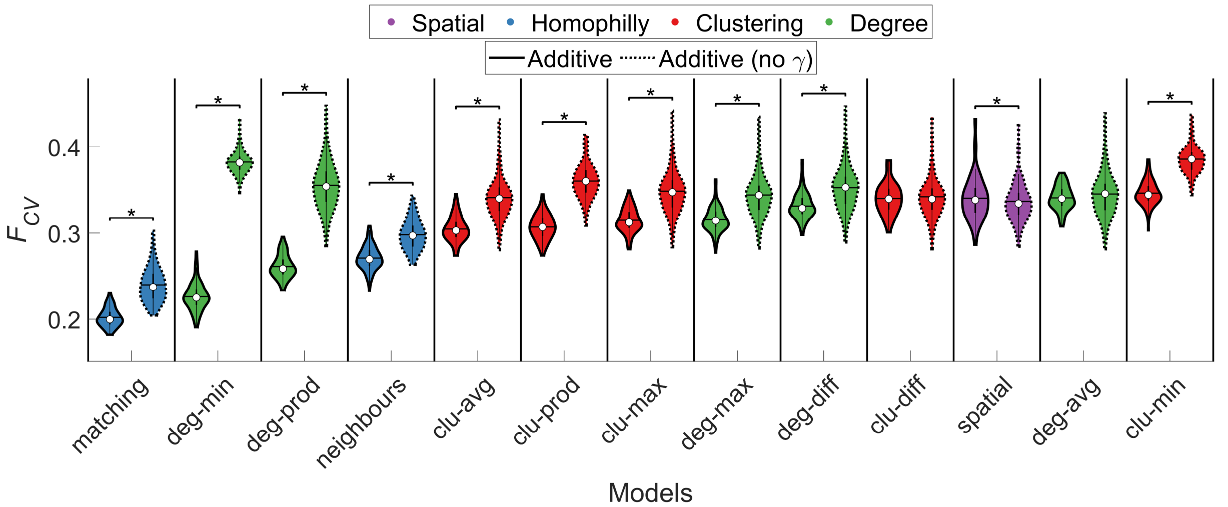

Fig. S3. **Model performance for growth additive generative trade-off models.** Each violin plot shows the distribution of cross-validated F_{CV} values for growth additive models with and without a γ parameter for the topology term across subjects. The color of each violin plot indicates the topology metric used in the model: homophily is shown in blue, clustering (clu) in red, degree (deg) in green, and spatial in purple. The white circle indicates the median of each distribution, while the horizontal black line indicates the mean. The matching growth model achieved the best performance. * $p_{FWER}(325) < .05$ (Wilcoxon signed-rank test, Bonferroni corrected for all 325 tests between all 26 models). Additive models with non-linear scaling of the topology term (i.e., with the γ parameter) produced the best performance.

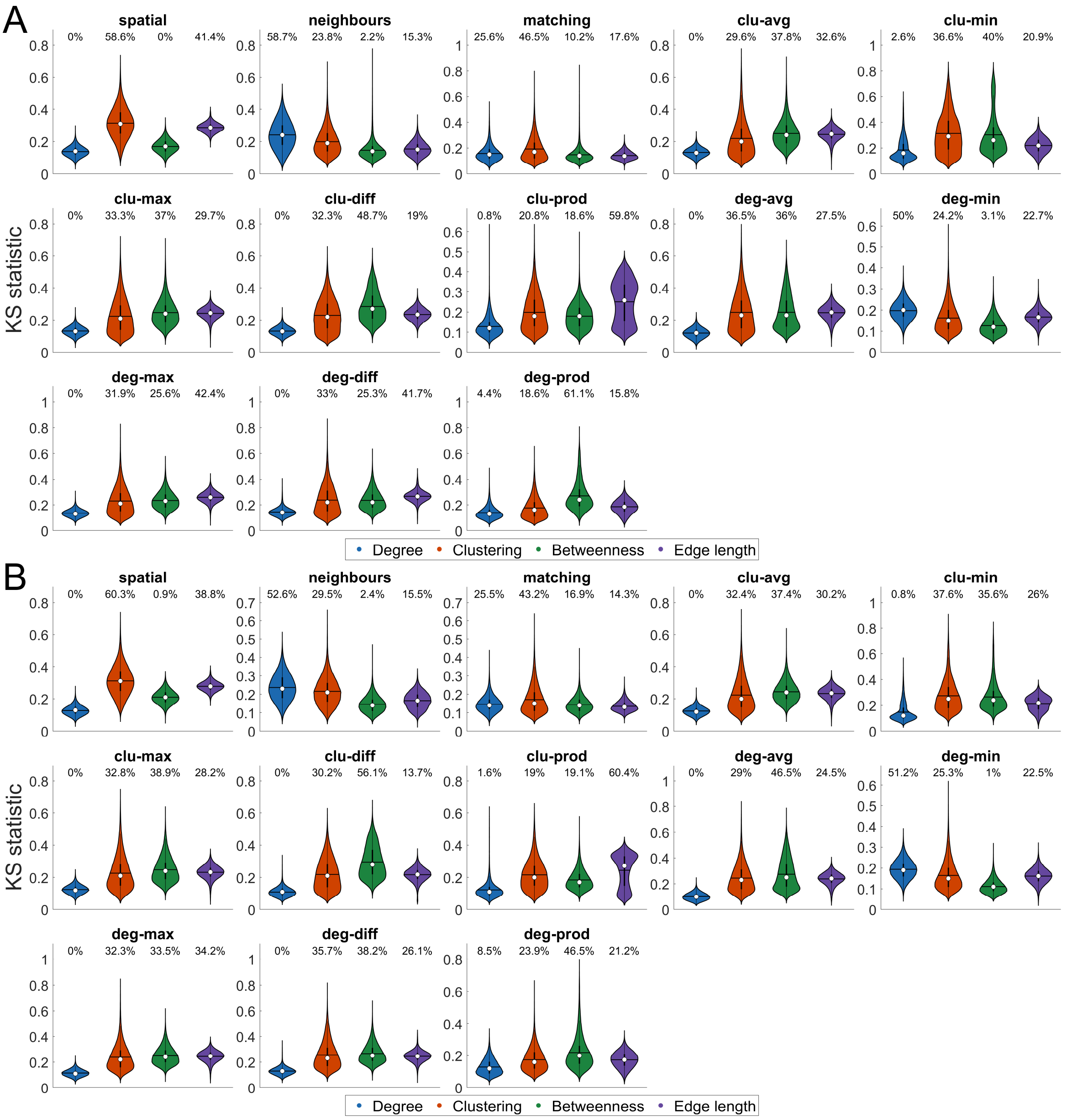

Fig. S4. **Kolmogorov-Smirnov (KS) statistics for each cross-validated static and growth model network.** Each violin plot shows the distribution of KS statistics across all model networks used to derive the cross validated fit F_{CV} for static (**A**) and growth (**B**) model variants, for each topological feature used in the model fitting procedure; namely, degree, clustering, betweenness, and edge length distributions. The white circle indicates the median of each distribution, while the horizontal black line indicates the mean. The number above each violin plot indicates the percentage of times that respective KS statistic determined model fitness i.e., $\max(KS)$.

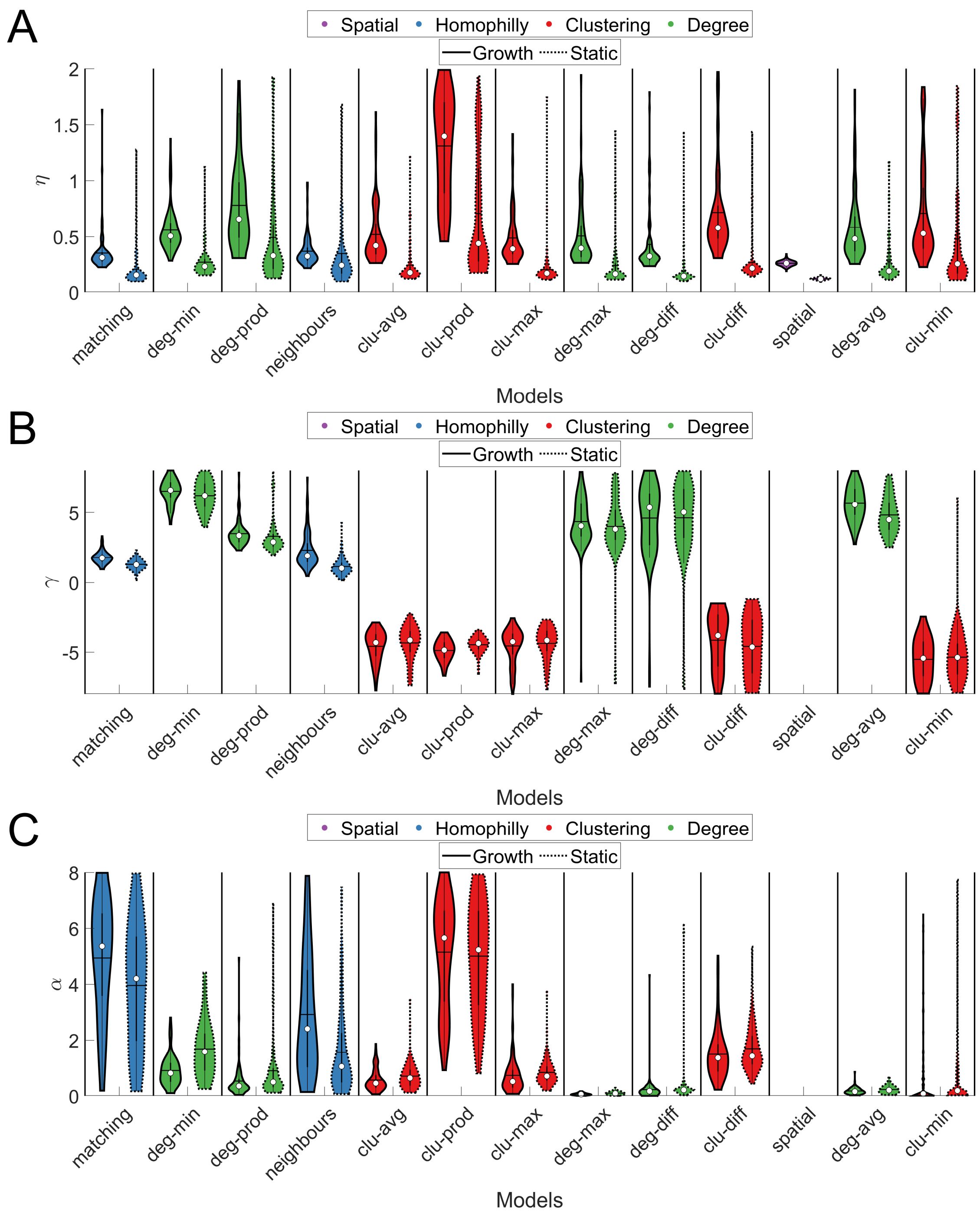

Fig. S5. **Model parameters of best-fitting static and growth models.** **(A)** Values of η. Higher values indicate a stronger distance penalty. **(B)** Values of γ. Higher values indicate a stronger non-linear scaling of topology, such that high values exert a proportionally greater influence on connection probability. **(C)** Parameters for α**.** Higher values indicate a stronger bias of topology relative to wiring cost on connection probability. The color of each violin plot indicates the class of topology metric used in the model: homophily is shown in blue, clustering in red, degree in green, and spatial in purple. The white circle indicates the median of each distribution, while the horizontal black line indicates the mean. Growth models require a larger η, and thus stronger distance penalty, to achieve the best fits.

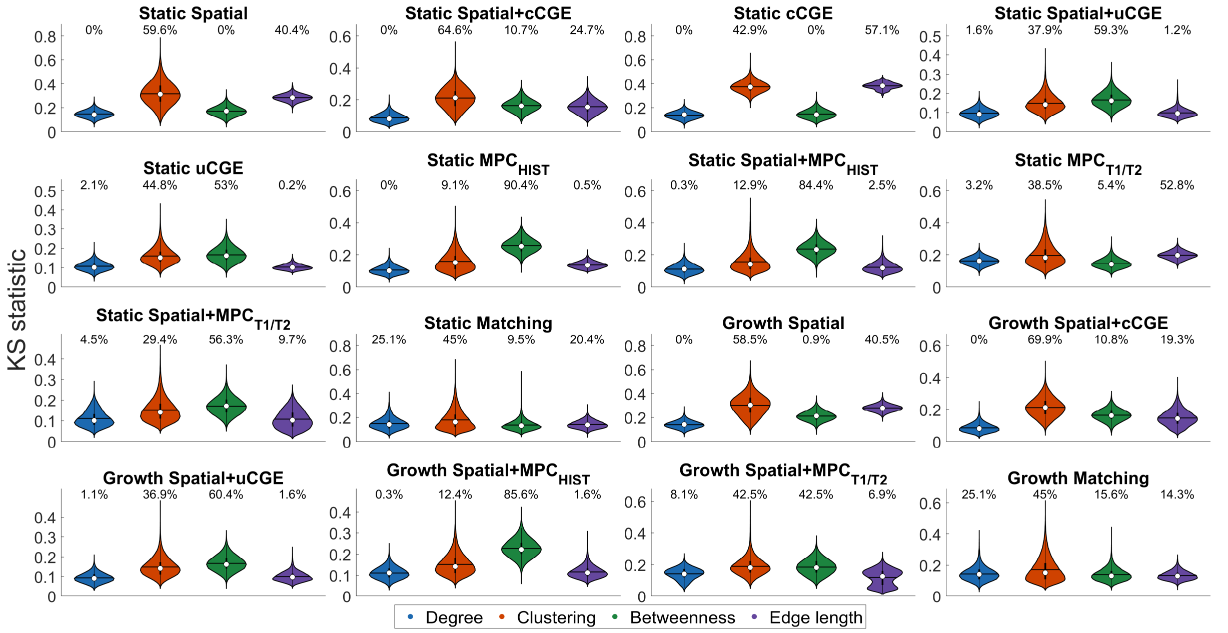

Fig. S6. **Kolmogorov-Smirnov (KS) statistics for each physiological model network.** Each violin plot shows the KS statistic for the relevant topological feature (node degree, node clustering, node betweenness, or edge length) for all networks used to calculate F_{CV} for each participant. The white circle indicates the median of each distribution, while the horizontal black line indicates the mean. The number above each violin plot indicates the percentage of times that KS statistic determined model fitness i.e., $\max(KS)$, which itself was used to derive the cross validated fit F_{CV}.

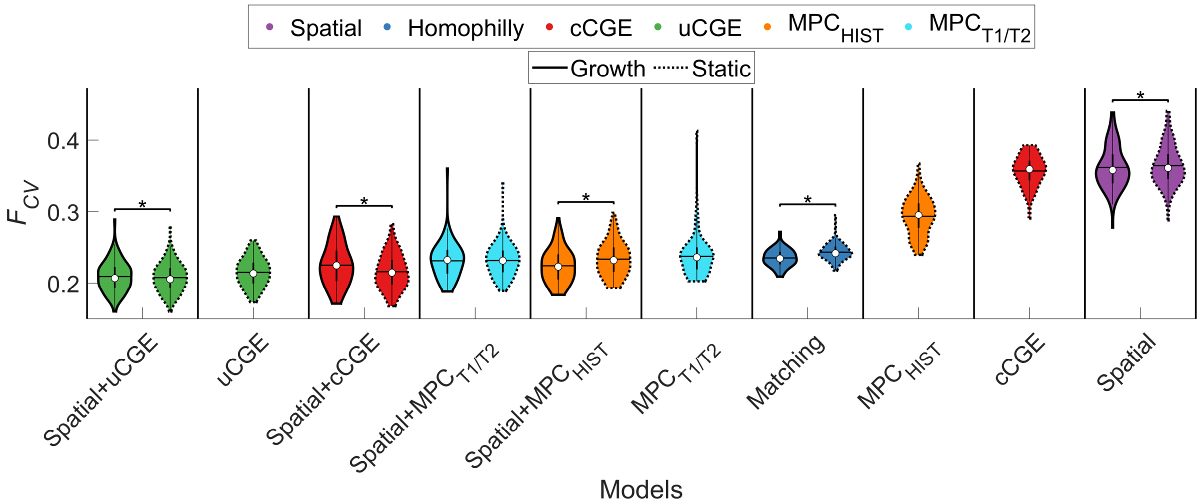

Fig. S7. **Model performance of physiologically-informed models for the Schaefer parcellation.** Each violin plots shows the F_{CV} values different models. The color of each violin plot indicates the type of model: matching is shown in blue, cCGE in red, uCGE in green, MPC_{HIST} in orange, $MPC_{T1/T2}$ in cyan, and spatial in purple. The white circle indicates the median of each distribution, and the horizontal black line indicates the mean. uCGE models achieved the best fit. Note that uCGE, cCGE, MPC_{HIST}, $MPC_{T1/T2}$ models do not have a growth variant as they do not have an independent distance term. * $p_{FWER}(120) < .05$ (Wilcoxon signed-rank test, Bonferroni corrected for all 120 tests between all 16 models).

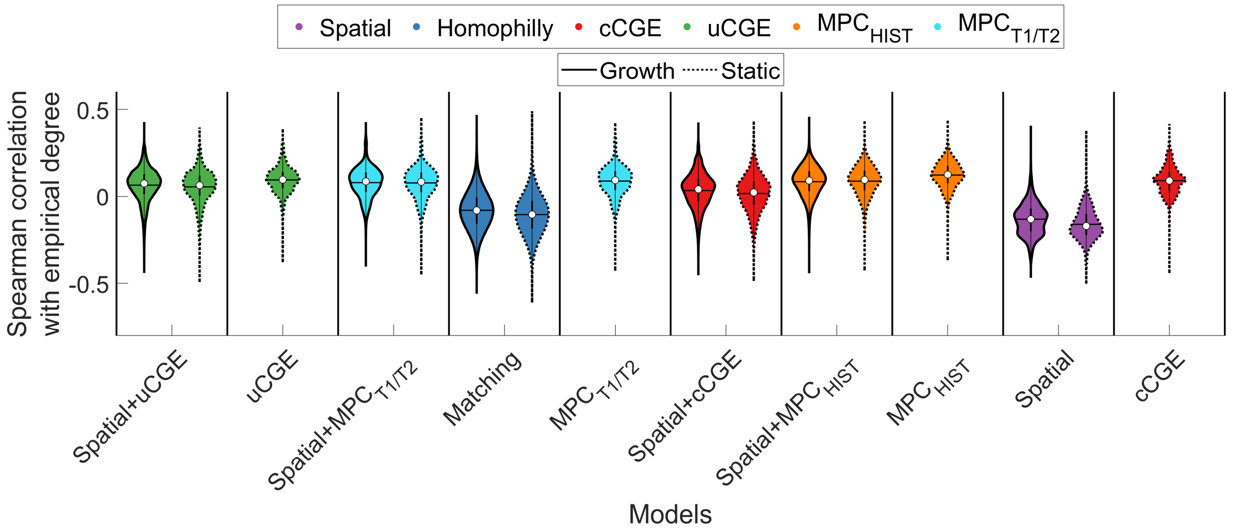

Fig. S8. **Evaluating model accuracy in capturing degree topography across the full parameter landscape.** Each violin plot shows, across all parameters produced during optimization for all participants, the Spearman correlation between model and empirical node degree. The color of each violin plot indicates the topology metric used in the model while the white circle indicates the median of each distribution, while the horizontal black line indicates the mean.

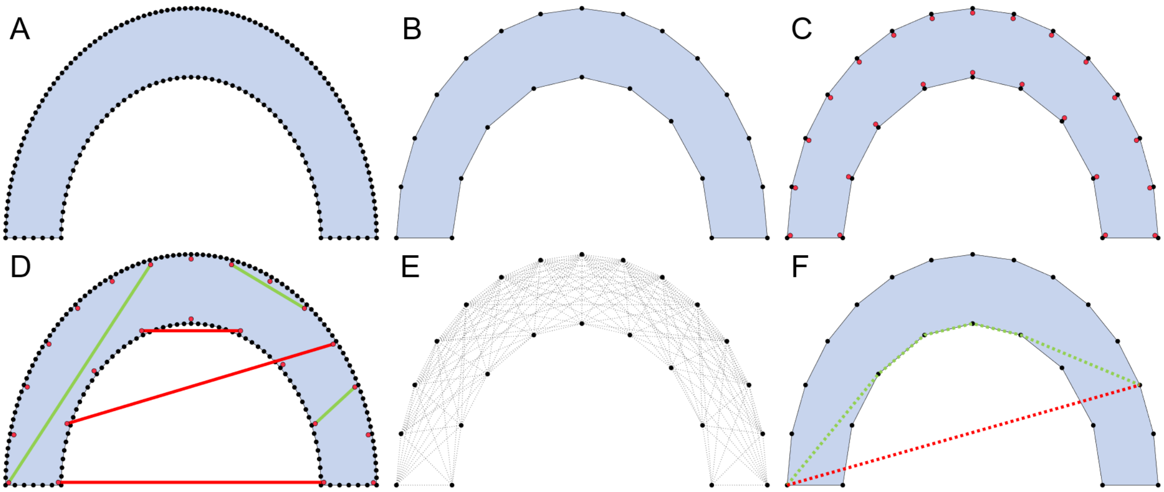

Fig. S9. **Example of connection distance estimation.** (**A**) Simplified example of a cortical surface reconstruction with black points representing surface vertices. The interior of the surface is shaded in light blue. (**B**) The surface is downsampled so that only 15% of the original vertices remain. This step minimizes computational burden while preserving the shape of the surface. (**C**) Subsurface points (red dots) are calculated using the vertex normal to avoid precision issues. (**D**) Ray tracing is used to draw line segments between pairs of subsurface points. Green lines show valid trajectories that do not intersect the surface. Red lines show invalid trajectories which are not used for further estimation. (**E**) All pairs of subsurface points are evaluated. If a valid line segment can be drawn between them, then a direct connection (dotted line) is drawn between the corresponding vertices to create a direct connection network. (**F**) Dijkstra's algorithm is run on the resulting network of pairwise direct connections (using the distances of each direct connection) to identify the shortest within-volume distance (i.e., fiber distance) between vertex pairs. The green line shows an example trajectory used to estimate fiber distance between two vertices. The trajectory used for estimating the Euclidean distance between those same points is shown by the red dotted line.
